# Effects of inspiratory muscle training in patients with obstructive sleep apnoea syndrome: a systematic review and meta-analysis

**DOI:** 10.5935/1984-0063.20220081

**Published:** 2022

**Authors:** Javid Ahmad Dar, Aqsa Mujaddadi, Jamal Ali Moiz

**Affiliations:** Jamia Millia Islamia, Centre for Physiotherapy and Rehabilitation Sciences - New Delhi - India

**Keywords:** Inspiratory Muscle Trainer, Inspiratory Muscle Training, Obstructive Sleep Apnoea, Obstructive Sleep Apnoea Syndrome

## Abstract

Obstructive sleep apnoea (OSA) is a common disorder marked by repetitive occurrence of breathing cessation during sleep due to partial or complete upper airway obstruction. An obstructive airway and the successive asphyxia chronically overload the inspiratory muscles resulting in an increased inspiratory effort. The present systematic review aimed to examine the effects of inspiratory muscle training (IMT) on inspiratory muscle strength [maximal inspiratory pressure (PImax)], severity of disease [apnea hypopnoea index (AHI)], sleep quality [Pittsburgh sleep quality index (PSQI)], day time sleepiness [Epworth sleepiness scale (ESS)], lung function [forced expiratory volume in 1 second (FEV_1_)] and exercise capacity [cardiopulmonary exercise testing, (CPET), 6 minute walk test, (6MWT)] in mild to severe OSA. Among 953 articles retrieved from various databases (PubMed, SCOPUS, Web of Science and Cochrane), 7 articles were found to be eligible for the present review. Randomized controlled trials reporting the effect of IMT in OSA were selected. The quality assessment was conducted using Cochrane risk-of-bias tool for randomized trials. All seven studies were meta-analyzed. The result depicted significant change in PImax, ES 1.73 (95%CI 0.54 to 2.92, *p*=0.004), PSQI -1.29 (95%CI -1.94 to -0.65, *p*<0.0001), ESS -1.08 (95% CI -1.79 to - 0.37, *p*=0.003) and FEV_1_ 0.74 (95%CI 0.20 to 1.28, *p*=0.007). IMT may be considered as an effective treatment strategy in mild to severe OSA resulting in improved inspiratory muscle strength, sleep quality, daytime sleepiness, and lung function. However, there is still dearth evidence on repercussion of IMT on lung function and exercise capacity and warrants high quality evidence to reach definitive conclusions.

## INTRODUCTION

The prevalence of obstructive sleep apnea (OSA) is recently reported to be around 9%-38%, reaching alarming levels^1^. OSA is a respiratory sleep disorder characterised by hypopnoea (partial) or apnoea (complete) resulting in occlusion of upper airway^2^. An occluded airway in OSA may stimulate an increased inspiratory effort which significantly lowers the functioning of inspiratory muscles^3^. Further, hypoxic episodes during sleep, may result in systemic manifestations including sleep fragmentation, excessive day time sleepiness, impaired sleep quality, and exercise capacity^4^.

The prevailing “gold standard” treatment for OSA is continuous positive airway pressure (CPAP). Apart from CPAP, surgical interventions, intraoral and nasal valve devices are generally considered for OSA treatment, but owing to its less cost-effectiveness and sophisticated implementation it results in reduced patient adherence as long-term management strategy^5-7^. In this context, considering respiratory burden and systemic manifestation in OSA, exercise training is well-tolerated adjunct treatment strategy. Recently conducted meta-analysis^8^ showed positive effect of exercise training in OSA.

Regarding inspiratory muscle training (IMT), a form of resistance training which improves the strength and performance of respiratory muscles in healthy individuals as well in patients with cardiorespiratory diseases^9^. Specific to the utilization of IMT in OSA there is significant literature gap^10^. Recently conducted investigations yields controversial findings with studies depicting significant improvement in inspiratory muscle strength (IMS), sleep quality, lung function, and apnea-hypopnoea index (AHI)^11-14^, while other studies depicted no significant change in lung function, AHI and exercise capacity^10,15,16^ following IMT. Hence the effect of IMT in OSA is debatable. Therefore, the aim of this systematic review and meta-analysis is to examine the effect of IMT on IMS, AHI, sleep quality, daytime sleepiness, lung function, and exercise capacity in people diagnosed with OSA.

## MATERIAL AND METHODS

The protocol for this systematic review is registered in the International Prospective Register of Systematic Reviews (CRD42020222138) on 19^th^ Nov 2020, before titles were investigated and selected for search results. This review is following Preferred Reporting Items for Systematic Reviews and Meta-Analyses (PRISMA) guidelines^17^.

### Eligibility criteria

We included only randomized controlled trials (RCTs) or randomized cross-over trial published in English Language; patients with a diagnosis of OSAS irrespective of AHI;IMT as a major intervention(s) with duration of 5-45 minutes. The intervention was administered in either institutional or home setting. The exclusion criteria of the study were patients who had history of pulmonary disease^18^. Patients having bipolar disorder, schizophrenia, uncontrolled hypertension, renal disease, and metabolic or endocrine disorders were excluded. Patients using CPAP, any recent neck surgeries, positive history for recurrent laryngeal spasm and lung surgery were also excluded from the present study.

### Search strategy and information sources

A systematic literature search was performed on the PubMed, Scopus, Web of Science and the Cochrane library for clinical trials. The search strategy was performed using the following key terms: “inspiratory muscle training”, “inspiratory muscle trainer”, “obstructive sleep apnoea”, “obstructive sleep apnoea syndrome”, “OSA”, and “IMT”. Using these key terms, exhaustive list of keywords was created to build the specific search strategy for each database. The list of keywords was generated through several steps in order not to miss relevant articles from specific search engines. This step was carried out through brainstorming sessions among research team members. An initial keywords plan and search strategy was conceptualised via expert consensus of the team members coupled with the gathering of previous literature. The search strategy in PubMed was built based on the research question formulation (i.e., PICO), i.e., “population” (obstructive sleep apnoea OR obstructive sleep apnoea syndrome OR OSAS) “intervention” (inspiratory muscle training OR inspiratory muscle trainer OR IMT). Additionally, we did not use “outcomes”, as their inclusion hindered the database being searched to retrieve eligible studies because the used outcome was not mentioned in the articles. Boolean operators “AND” and “OR” were used to connect key terms to obtain more focused and productive results. Besides, electronic database the reference list of all primary articles were screened and reviewed for additional references and the study authors were contacted for any missing information.

### Study selection

**Figure 1:**
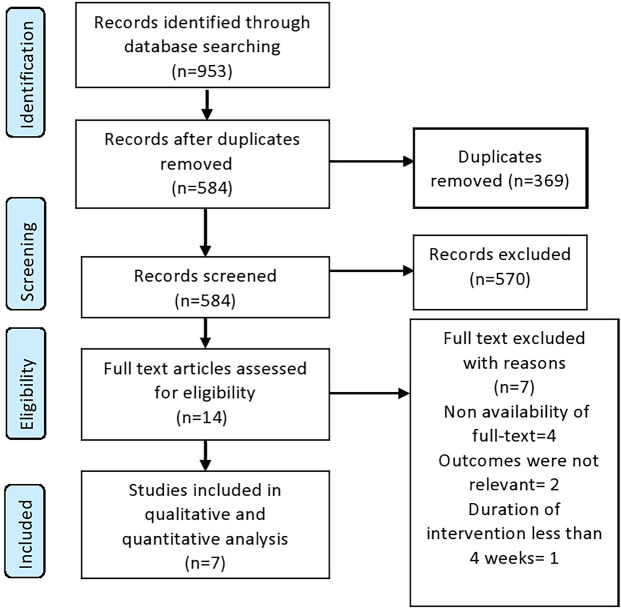
PRISMA flow diagram of the included studies.

The studies which met all the inclusion and exclusion criteria and were relevant to the effects of IMT in OSA patients were taken into consideration by the two authors (J.A.D and A.M) (Figure 1). The duplicates were removed from the searched articles and the selected articles were screened at the title/abstract stage and the full-text stage for eligibility. In case of any disagreement, it was resolved through discussion and if needed a third reviewer was contacted (J.M).

### Data extraction

The data was extracted from each article comprising general information (author, publication date, country, experimental dates), study characteristics (study design, duration), the participants (sample size, age, sex), intervention (type, intensity, frequency, duration, number of sessions, supervision), control treatment (Table 1). Primary outcome measures (IMS, AHI, sleep quality, daytime sleepiness) and secondary outcomes (lung function and exercise capacity) and the main findings were extracted by the two authors independently (J.A.D and A.M.). If the reported data were incomplete or unclear, authors of that study were contacted. For meta-analysis, descriptive data, i.e., mean and standard deviation (SD) of the relevant outcome measures, were recorded. Any conflicts between the reviewers were resolved by consensus with a third reviewer (J.M.)

**Table 1:** Characteristic of studies included in the systematic review.

Study/year	Country	Method	Sample size (N=at baseline;	Patient characteristics (mean age in years) male %	Severity of OSAS (AHI) mild (5-14.9/hour), moderate (15-29.9/hour) and severe(≥30/hour)	Mode of IMT and supervision	Time, intensity and progression of IMT	Frequency and duration of IMT	Monitoring of Breathing Pattern	Outcomes assessed, Findings
Moawd et al., 2020^15^	Egypt	Training group (IMT *versus* placebo training group (P-IMT)	55	57 years (36%)	Mild to moderate OSA.	Targeted inspiratory resistance trainer (resistance trainer with visual feedback) supervised	30 min a session @ 0-300cmH_2_O. @75% of PImax.	three times a week for 12 consecutive weeks.	six cycles of thirty breaths.	Inspiratory Muscle Strength ↑­ AHI ↑ Lung Functions ↑ Aerobic Capacity ↑
Vranish and Bailey, 2016^10^	United States	IMT training *versus* placebo IMT	24	65 years (81.8%)	Mild to moderate and severe OSA	Inspiratory threshold training device supervised	5 min session @ 75% of PImax.	Once a day for 6 weeks	30 breaths each day for 6 w	Inspiratory Muscle Strength, ↑ AHI ↑ Sleep and Sleep Quality↑
Erturk et al., 2020^12^	Turkey	(IMT) *versus* OE versus control	54	49 years (76%)	Mild to moderate and severe OSA	Threshold loading device supervised	15min session @ 30% of MIP	twice a day, 7 days/week for 12 weeks	4-5 controlled breaths.	Inspiratory muscle strength, ↑ AHI ↑­ Exercise capacity ↑ Sleep quality ↑
Andhare et al., 2020^11^	India	IMT threshold device *versus* control group	145	51 years (25%)	Mild to moderate and severe OSA (Stop-Bang Questionnaire)	Inspiratory threshold training device supervised	5 minutes session @ 60-80% of 1 RM	Once 3 days/week for 4 weeks	3 controlled breaths.	Inspiratory muscle strength, ↑ Sleep quality ↑ AHI ↑
Lin et al., 2020^13^	Taiwan	TIMT group, *versus* TIMT; control group medical treatment and routine care, but no TIMT	22	53 years (62.5%)	Moderate to severe OSA	TIMT device. home-based TIMT	30-45min. session @11 and 21cmH_2_O; weekly pressure increase was 5%;	twice 5 days/week, for 12 weeks	3 -4 controlled breaths.	Inspiratory muscle strength, ↑ AHI ↑ ­ Sleep quality ↑­ Lung Functions ↑­ Daytime sleepiness ↓
Souza et al., 2018^16^	Brazil	IMT *versus* placebo P-IMT	30	52 years (66.6%)	Moderate to severe OSA	Inspiratory muscle trainer Home as well as lab (quarterly) supervised	15 minutes session@50-60% of MIP	twice a day 7 days a week, For 12weeks	3 controlled breaths.	Inspiratory muscle strength, ↑ ­ AHI ↑ ­ Exercise capacity - Sleep quality ↑­ Lung Functions - Daytime sleepiness ↓
Nobrega et al., 2020^14^	Brazil	IMT versus placebo P-IMT	35	59 years (50%)	Moderate or severe OSA	Powerbreath IMT, supervised	15 minutes session@50-75% of MIP	twice a day 7 days a week, For 8weeks	3 cycles of 30 breaths	Inspiratory muscle strength, ↑ AHI ↑ ­ Exercise capacity ↓ Sleep quality ↑ Lung Functions - Daytime sleepiness ↓

OSA = Obstructive sleep apnea; IMT = Inspiratory muscle training; TIMT = Threshold inspiratory muscle training; P-IMT = Placebo inspiratory muscle training; CPET = Cardiopulmonary exercise testing; MIP = Maximal inspiratory pressure; AHI = Apnea hypoapnea index; RM = Repetitive maximum; PEFR = Peak expiratory flow rate; PSQI = Pittsburgh sleep quality index; ESS = Epworth sleepiness scale; OE = Oropharyngeal exercises. ↑ = Increased; ↓ = Decreased; - = No change

### Risk of bias

**Figure 2:**
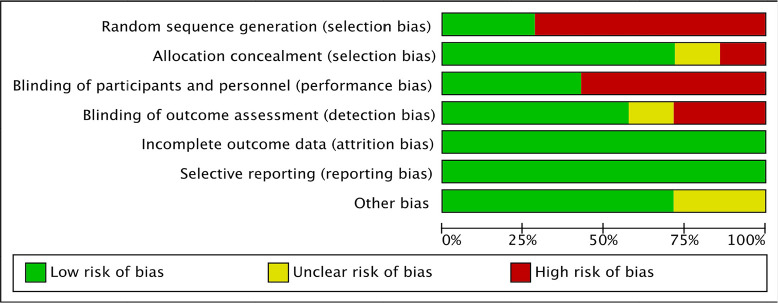
Risk of bias graph: review authors judgement about each risk of bias item presented as percentages across all studies.

Two authors (J.A.D and A.M) independently assessed the risk of bias of each individual study using Cochrane Risk of Bias tool 2 (RoB 2) for RCT against key criteria^19^. The domains included randomization process, deviations from intended interventions, selection of the reported result, measurement of the outcome and overall bias. The following judgements were used: low risk, high risk, or unclear (either lack of information or uncertainty over the potential for bias)^20^. The risk of bias of included studies was summarised for each domain (Figure 2). Any disagreements were resolved through discussion or consulting third author (J.M.) if necessary.

### Quality of evidence: GRADE-criteria

We performed the overall quality of the evidence applying the GRADE approach as advised by the Cochrane Handbook for Systematic Reviews of Interventions^21^. As for each specific outcome, the quality of the evidence was obtained based on 5 factors: (1) limitations of the study design; (2) consistency of results; (3) directness; (4) precision, and (5) potential for publication bias. The quality was reduced by one level for each of the factors not satisfied. The GRADE approach followed in 4 levels of quality of evidence: high, moderate, low, and very low^22^. GRADE profiler software was used to rate the quality of evidence^23^. The overall quality of evidence in this systematic review was low-moderate (Table 2) due to the high risk of bias, as most of the RCTs were not double blinded, majority were lacking in allocation concealment and also due to heterogeneity in the data.

**Table 2:** GRADE approach to assess quality of evidence.

Summary of findings:				
Effect of inspiratory muscle training compared to placebo IMT for on obstructive sleep apnoea syndrome.				
Patient or population: on obstructive sleep apnoea syndrome Setting: Hospital/Home Intervention: effect of inspiratory muscle training Comparison: placebo IMT				
Outcomes	Anticipated absolute effects^^*^^ (95% CI)			
	Risk with effect of inspiratory muscle training	№ of participants (studies)	Certainty of the evidence (GRADE)	Comments
Inspiratory muscle strength assessed with: PImax cmH_2_O follow-up: range 4 weeks to 12 weeks	SMD 1.73 SD higher (0.54 higher to 2.92 higher)	273 (6 RCTs)	⨁⨁⨁◯ Moderate^a,b^	Effect of inspiratory muscle training probably results in a large increase in inspiratory Muscle Strength.
Apnoea hyponea index (AHI) follow-up: range 4 weeks to 12 weeks	SMD **0.11 SD lower** (0.49 lower to 0.28 higher)	102 (4 RCTs)	⨁⨁⨁◯ Moderate^a,b^	The evidence suggests effect of inspiratory muscle training reduces apnoea hyponea Index.
Sleep quality assessed with: PSQI scale from: 0 to 3 follow-up: range 4 weeks to 12 weeks	SMD **1.29 SD lower** (1.94 lower to 0.65 lower)	227 (6 RCTs)	⨁⨁⨁◯ Moderate^a,b^	The evidence suggests effect of inspiratory muscle training reduces sleep quality slightly.
Day time sleepiness assessed with: ESS scale from: 0 to 24 follow-up: range 4 weeks to 12 weeks	SMD **1.08 SD lower** (1.79 lower to 0.37 lower)	103 (4 RCTs)	⨁⨁◯◯ Low^a,b^	Effect of inspiratory muscle training may result in a slight reduction in day Time Sleepiness.
Lung function assessed with: FEV1 follow-up: range 4 weeks to 12 weeks	SMD **0.74 SD higher** (0.2 higher to 1.28 higher)	86 (3 RCTs)	⨁⨁◯◯ Low^a,b^	Effect of inspiratory muscle training may increase/have little to no effect on lung Function but the evidence is very uncertain.
Exercise capacity assessed with: VO_2_, follow-up: range 4 weeks to 12 weeks	SMD **0.24 SD higher** (0.6 lower to 1.07 higher)	98 (3 RCTs)	⨁⨁◯◯ Low^a,b^	The evidence suggests effect of inspiratory muscle training results in a slight reduction in exercise capacity.

Notes: CI = Confidence interval; SMD: Standardised mean difference; ESS: Epworth sleepiness scale; AHI: Apnoea hyponea index; PSQI = Pittsburgh sleep quality; FEV1 = Forced expiratory volume in one second; Explanations - a. Blinding was missing, randomisation process was not mentioned mostly; b. The method of study, frequency, and duration of intervention was different that can lead to heterogeneity.

## RESULTS

A total of 953 articles were identified (n=261, Scopus), (n=185, Web of Science), (n=471, PubMed), and (n=36, Cochrane database), of which 369 were duplicates. After screening 584 records, 14 articles were found to be eligible for full text-evaluation. Of those, 7 were excluded, and 7 articles met inclusion criteria and included for qualitative and quantitative analysis.

The mean age of the participants reported across studies was 49-65 years. Five studies clinically diagnosed OSA by polysomnography (PSG)^10,12,13,15,16^ while two studies^14,11^ utilised Berlin and STOP BANG questionnaire, respectively. The severity of OSA was diagnosed using AHI as mild (5-14.9/hour), moderate (15-29.9/hour), and severe (≥30/hour)^24^.

IMT is a form of resistance training, which strengthens pharyngeal, intercostals, and diaphragm musculature while allowing these muscles to be trained as a group against a specific resistance^10^. IMT was delivered with threshold inspiratory muscle trainer device (TIMT) in four studies^10-13^. Two studies^14,16^ delivered IMT through power breathe classic light device. One study^15^ used electronic inspiratory muscle trainer (TRAINAIR, UK). Five studies^10,13-16^ included IMT as the sole intervention with respiratory pressure ranging from 30%-75% of PImax while other two studies^11,12^ added oropharyngeal and conventional breathing exercises to IMT. The placebo IMT was administered to the comparison group with pressure ranging from 0%-15% of PImax. Each IMT session time ranges from 5-45 minutes maximum and weekly session ranging from once a week for four weeks to thrice a week for twelve weeks.

The primary outcome measure was IMS^10-12,14-16^, AHI^10,12-14^, sleep quality^10-14,16^ and day time sleepiness^12-14,16^. The secondary outcome measure included lung function^13,15,16^ and exercise capacity^12,15,16^. IMS is mostly measured by the PImax in patients with respiratory muscle weakness, PImax diagnose inspiratory muscle weakness earlier than change in lung volumes^25^. AHI was evaluated by PSG in the sleep laboratory^26^. Sleep quality was assessed from the Pittsburgh sleep quality index (PSQI)^27^. The total score >5 in PSQI indicates poor sleep quality. The Epworth sleepiness scale (ESS) was used to assess daytime sleepiness^28^. A total score >10 in ESS indicated significant daytime sleepiness. Lung function was assessed using the standard spirometry where patients held three deep breaths, and seated with flexed knees at 90°, inspired up to the total lung capacity (TLC) and then exhaled all the air to their residual volume (RV) to obtain the variables FEV_1_ (forced expiratory volume in 1 second), PEF (peak expiratory flow), FVC (forced vital capacity) and FEV_1_/FVC. It was based on the guidelines of the American Thoracic Society (ATS)^25^. The exercise capacity was assessed by the cardiopulmonary exercise testing (CPET)^29^ or six minute walk test (6MWT)^12^ in patients with OSA.

### Risk of bias in included studies

**Figure 3:**
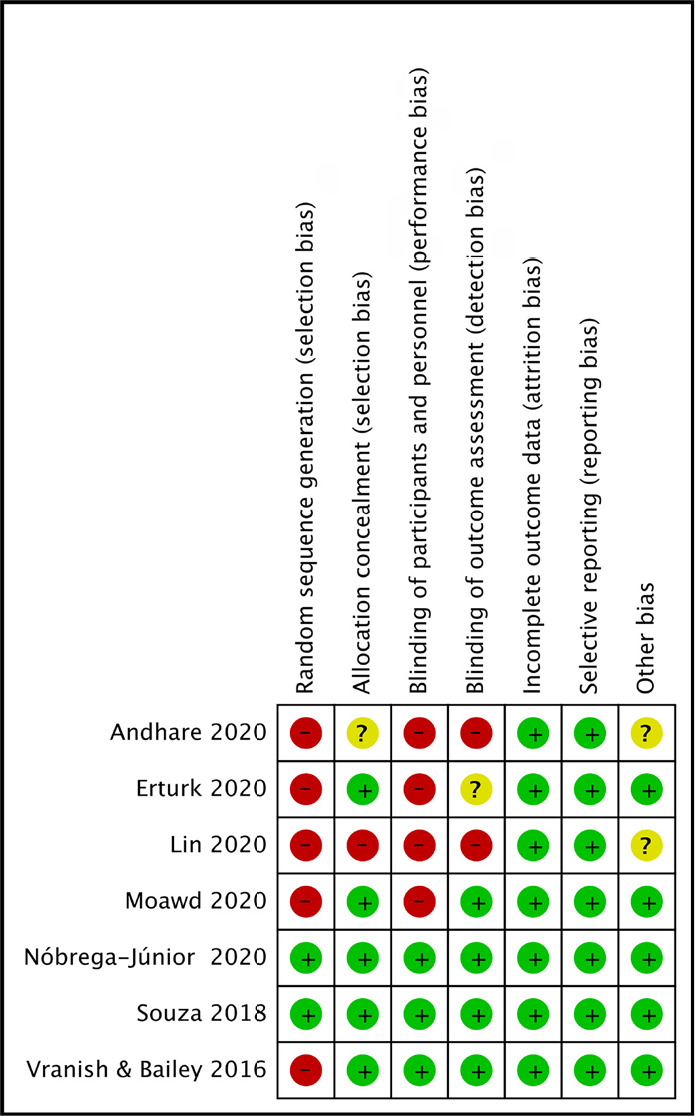
Risk of bias summary: review authors’ judgement about each risk of bias item for each included study.

Seven studies^10-16^ were included in which only two^14,16^ had low risk of bias as method of randomization process was described and the remaining five studies^10-13,15^ reported as high risk of bias as there was lack of detail in the randomization method. The five studies^10,12,14-16^ had low risk of bias in allocation concealment (envelope method, telephone service) one had a higher risk^13^ and in one study it was unclear^11^. Three studies^10,14,16^ were of lower risk because of participant’s blinding while remaining four^11-13,15^ had higher risk of bias due to lack of blinding. The selective reporting data and incomplete data were of low risk in all the selected seven studies^10-16^ in both the domain. Five studies^10,12,14-16^ were of lower risk due to the placebo, whereas the two studies^11,13^ the risk of bias was unclear (Figure 3).

### Meta-analysis

Meta-analysis was performed using Review Manager 5.4 software by pooling data across studies for each outcome measure. Post-intervention mean and SD were used for meta-analysis through which pooled standardized mean differences (SMD) was computed between the intervention and comparator group. The chi-square test for heterogeneity was significant for IMS (*p*<0.00001), sleep quality (*p*=0.002), and exercise capacity (*p*=0.03). Using the random effect model, I^2^ value was a 93% for IMS, 73% for sleep quality, and 73% for exercise capacity. The I^2^ value suggests study variability (i.e., heterogeneity) in quantitative analysis. Low heterogeneity is depicted by value less than 25%, moderate is reflected by 25-50%, >75% reflects high heterogeneity. The heterogeneity of the studies could be due to the methodological aspects such as study quality or length of follow-up. It could also be due to the clinical aspects such as age, sex, co-morbidities and differences in interventions.

### Synthesis of results

#### Inspiratory muscle strength (IMS)

**Figure 4:**
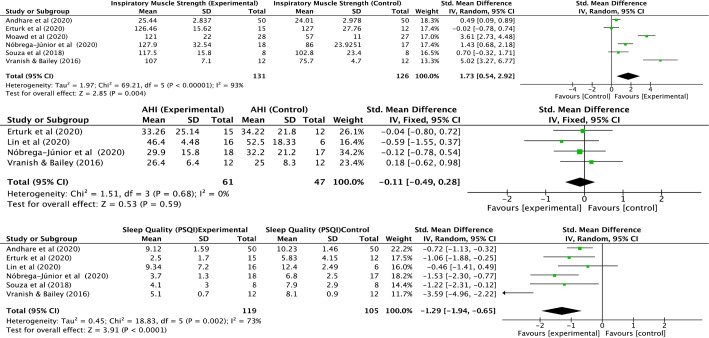
Meta-analysis of inspiratory muscle strength, apnoea-hypoapnea index and sleep quality.

In six studies^10-12,14-16^ PImax (cmH_2_O) was used to measure IMS while one study^13^ reported it as 1 repetition maximum (1RM). The six studies included in meta-analysis, constituted a total of 257 participants. The meta-analysis indicated significant large change in SMD, 1.73 (95%CI 0.54 to 2.92, *p*=0.004) to the IMT in patients with OSA (Figure 4).

#### Apnoea hypoapnea index (AHI)

Four studies^10,12-14^ were included in the meta-analysis with 108 participants reporting insignificant change following IMT with SMD -0.11 (95%CI -0.49 to 0.28, *p*=0.59) (Figure 4).

#### Pittsburgh sleep quality index (PSQI)

Sleep quality was analysed quantitatively in six studies^10-14,16^ including 224 participants. The SMD was large in experimental group following IMT -1.29 (95%CI -1.94 to -0.65, *p*<0.0001) (Figure 4).

#### Epworth sleepiness scale (ESS)

**Figure 5:**
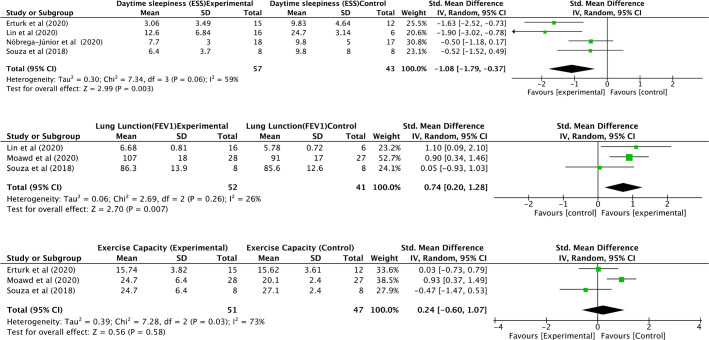
Meta-analysis of daytime sleepiness, lung function and exercise capacity.

The four studies^12-14,16^ were included in the meta-analysis of the same with 100 participants. The quantitative analysis depicted significant large improvement in ESS scores with SMD-1.08 (95%CI -1.79 to -0.37, *p*=0.003) in response to the IMT in OSA (Figure 5).

#### Lung function

Only 3 studies^13,15,16^ were used in the meta-analysis incorporating FEV_1_ with a total of 93 participants. There SMD reported was moderate and significant, 0.74 (95% CI 0.20 to 1.28, *p*=0.007) (Figure 5).

#### Exercise capacity

Three studies^12,15,16^ were included in the quantitative analysis with 98 subjects. The meta-analysis depicted insignificant improvement in the exercise capacity with a SMD of 0.24 (95% CI -0.60 to 1.07, *p*=0.58) following IMT (Figure 5).

## DISCUSSION

This systematic review and meta-analysis evaluated the effect of IMT on IMS, AHI, sleep quality, daytime sleepiness, lung function, and exercise capacity in patients with OSA. The methodological quality of analysed studies is low to moderate which was evaluated utilising the GRADE approach. All the seven studies (RCTs) were included in meta-analysis, which supports IMT as a means to improve IMS, sleep quality, daytime sleepiness, and lung function in OSA. However, the results must be extrapolated in the light of caution due to high risk of bias and heterogeneity in the included studies. Furthermore, the evidence for lung function and exercise capacity was reported in limited studies and the change in AHI was insignificant. There has been no previous systematic review reporting the effect of IMT in patients with OSA.

The CPAP is considered the gold standard treatment for OSA patients be it mild, moderate or severe as expressed in the Cochrane collaboration published review^30^ and also a systematic review published by the National Institutes of Health Research (NIHR)^31^. However, the IMT protocol depicted a cost-effective and simple adjunct treatment for OSA patients who are reluctant or unable to tolerate CPAP. Studies^10-16^ utilising frequency and intensity of IMT were highly variable from 2 times/week to 4 times/week at 30%-75% of PImax, respectively. This highlights the need for clear guidelines about the IMT exercise program and its dosage. However, Gloeckl et al. (2013)^32^ has provided useful suggestions for the implementation of IMT during pulmonary rehabilitation.

The findings of meta-analysis showed improvement in IMS following IMT which supports the notion put forward by joint statement of American College of Chest Physicians (ACCP) and American Association of Cardiovascular and Pulmonary Rehabilitation (AACVPR) evidence-based guidelines that IMT might be considered in patients with reduced IMS^33^. The findings are also in consensus with Corrêa et al. (2011)^34^ in Type 2 diabetes mellitus (T2DM) patients and one of the published systematic review which stated that IMT delivered through inspiratory pressure threshold device (IPTL) significantly enhances IMS and endurance in adults with stable COPD^35^. In patients with OSA repetitive inspiratory effort against an obstructed airway may induce deleterious effects on the inspiratory muscles^3^. The mechanism of improved IMS following IMT could be due to the inclusion of resistance training with IPTL, which is primarily based on principle of overload and neural adaptation^3^.

Four studies^10,12-14^ were included in the meta-analysis which recorded insignificant changes in AHI to IMT. Mohamed et al. (2017)^36^ found a significant reduction in AHI at the end of 6 weeks of oropharyngeal exercise therapy in stroke patients with moderate OSAS. They found that this improvement was associated with increased retropalatal distance and a decrease in soft palate length, indicating improvement in pharyngeal morphology. The insignificant change observed in the present meta-analysis might be due to varying OSA severity in the included studies^10,12-14^.

Sleep quality was analysed quantitatively in the six studies^10-14,16^ which showed a significant change in PSQI scores post IMT. In this study^10^ there were no changes in ESS scores after the intervention, but it has revealed significant improvement on global PSQI score, sleep quality, and sleep duration. The respiratory events are generally considered to be a major cause for hypoxaemia and hypercapnia during sleep. These changes are responsible for stimulating the central and peripheral chemoreceptor’s which increases sympathetic nervous system drive and consequently cause sudden awakenings and arousals to restore ventilation^3^. This compensatory mechanism is responsible for sleep fragmentation and consequently a decrease in sleep quality^3^. IMT enhances sleep quality, reduces blood pressure and circulating plasma catecholamine’s in adults with OSA^10^.

Only 3 studies^13,15,16^ were used in the meta-analysis incorporating FEV_1_ revealing significant large change in the lung function following IMT. These findings were supported by Enright et al. (2004)^37^ which has demonstrated that IMT improves lung function in adults with cystic fibrosis. The intrathoracic pressure (ITP) generated during IMT is almost similar during OSA. During IMT stimulus happen when one is awake and well-oxygenated while in OSA, ITP swings during sleep which is the typical characteristic of OSA leading to hypoxaemia^10^. The improved FEV_1_ following IPTL is due to the activation of the diaphragm to a greater extent by allowing increased motor unit recruitment of inspiratory muscles, thereby allowing larger air to enter inside the lungs. Therefore, the effects of IMT might be more analogous to traditional forms of aerobic exercise on lung function^13^.

Four studies^12-14,16^ were incorporated in meta-analysis of ESS which revealed significant large change following IMT. Two studies^14,16^ utilized PowerBreathe classic light device to deliver IMT at 50-60% of PImax and 75% of PImax, respectively, while other two studies^12,13^ delivered TIMT through threshold device at 11-21% and 30% of PImax, respectively. Despite in variations with the protocol utilized to deliver IMT all studies showed improvement in ESS. This significant change after IMT could be attributable due to a lower baroreflex sensitivity and greater activation of the sympathetic nervous system associated with sleep arousals^38^.

Three studies^12,15,16^ were included in the quantitative analysis of the exercise capacity through maximal oxygen consumption (VO_2_max) estimation. The meta-analysis reported no significant improvement in the exercise capacity following IMT. Previous literature confirms that acute and chronic hypoxia leads to reduction in VO_2_ max. The decrease is reported to be directly proportional to drop in haemoglobin saturation^39^. The fact that IMT is restricted to respiratory musculature which might not result in sufficient physiological overload on the cardiovascular system in order to provide sufficient improvement in VO_2_ max^40^. In consensus with the findings of this study Edward (2013)^41^ also reported no alterations in ventilatory variables of CPET in healthy subjects.

### Clinical implications

This is the first systematic review and meta-analysis to assess the effect of IMT in patients with OSA till date. The evidence of short-term symptom relief with IMT is good although the data on longer-term health benefits is limited. The results of this review suggest justification to the assessment of clinical and cost effectiveness of IMT treatment in terms of long-term effects in OSA severity, in addition to the relief of symptoms. The side-effect profiles of IMT and other treatment options are not well documented in clinical trials. Further work should explore the preference and withdrawal from such trials, which would inform the tolerability of the treatment.

### Limitations

The included studies in this meta-analysis were heterogeneous, small sample size; few of low-quality evidence, higher risk of bias, and short term follow-up.

## CONCLUSION

This study concludes that IMT significantly improves the IMS, sleep quality, daytime sleepiness and lung function. Although deriving a definitive conclusion would be difficult at this stage due to high risk of bias and heterogeneity observed in the included studies.
